# The associations between dietary fibers intake and systemic immune and inflammatory biomarkers, a multi-cycle study of NHANES 2015–2020

**DOI:** 10.3389/fnut.2023.1242115

**Published:** 2023-08-31

**Authors:** Xiangjun Qi, Yanlong Li, Caishan Fang, Yingying Jia, Meicong Chen, Xueqing Chen, Jie Jia

**Affiliations:** ^1^The First Clinical School of Guangzhou University of Chinese Medicine, Guangzhou, China; ^2^Hospital of Chengdu University of Traditional Chinese Medicine, Chengdu University of Traditional Chinese Medicine, Chengdu, China; ^3^Department of Gynecology, Zhengzhou Second Hospital, Zhengzhou, China; ^4^Guangzhou First People’s Hospital, Guangzhou, China; ^5^Department of Laboratory Medicine, The First Affiliated Hospital of Guangzhou University of Chinese Medicine, Guangzhou, China; ^6^Department of Ultrasound, The First Affiliated Hospital of Guangzhou University of Chinese Medicine, Guangzhou, China

**Keywords:** dietary fiber, National Health and Nutrition Examination Survey, systemic immune-inflammation index, systemic inflammation response index, red blood cell distribution width-to-albumin ratio

## Abstract

**Background:**

In recent years, there has been considerable growth in abnormal inflammatory reactions and immune system dysfunction, which are implicated in chronic inflammatory illnesses and a variety of other conditions. Dietary fibers have emerged as potential regulators of the human immune and inflammatory response. Therefore, this study aims to investigate the associations between dietary fibers intake and systemic immune and inflammatory biomarkers.

**Methods:**

This cross-sectional study used data from the National Health and Nutrition Examination Survey (2015–2020). Dietary fibers intake was defined as the mean of two 24-h dietary recall interviews. The systemic immune-inflammation index (SII), systemic inflammation response index (SIRI), neutrophil-to-lymphocyte ratio (NLR), platelet-lymphocyte ratio (PLR), red blood cell distribution width-to-albumin ratio (RA), ferritin, high-sensitivity C-reactive protein (hs-CRP), and white blood cell (WBC) count were measured to evaluate systemic immune and inflammatory states of the body. The statistical software packages R and EmpowerStats were used to examine the associations between dietary fibers intake and systemic immune and inflammatory biomarkers.

**Results:**

Overall, 14,392 participants were included in this study. After adjusting for age, gender, race, family monthly poverty level index, alcohol consumption, smoking status, vigorous recreational activity, body mass index, hyperlipidemia, hypertension, diabetes, and dietary inflammatory index, dietary fibers intake was inversely associated with SII (β = −2.19885, 95% CI: −3.21476 to −1.18294, *p* = 0.000248), SIRI (β = −0.00642, 95% CI: −0.01021 to −0.00263, *p* = 0.001738), NLR (β = −0.00803, 95% CI: −0.01179 to −0.00427, *p* = 0.000284), RA (β = −0.00266, 95% CI: −0.00401 to −0.00131, *p* = 0.000644), ferritin (β = −0.73086, 95% CI: −1.31385 to −0.14787, *p* = 0.020716), hs-CRP (β = −0.04629, 95% CI: −0.0743 to −0.01829, *p* = 0.002119), WBC (β = −0.01624, 95% CI: −0.02685 to −0.00563, *p* = 0.004066), neutrophils (β = −0.01346, 95% CI: −0.01929 to −0.00764, *p* = 0.000064). An inverse association between dietary fibers and PLR was observed in the middle (β = −3.11979, 95% CI: −5.74119 to −0.4984, *p* = 0.028014) and the highest tertile (β = −4.48801, 95% CI: −7.92369 to −1.05234, *p* = 0.016881) and the trend test (β_trend_ = −2.2626, 95% CI: −3.9648 to −0.5604, *P_trend_* = 0.0150). The observed associations between dietary fibers intake and SII, SIRI, NLR, RA, ferritin, hs-CRP, WBC, and neutrophils remained robust and consistent in the sensitivity analysis. No significant interaction by race was found.

**Conclusion:**

Dietary fibers intake is associated with the improvement of the parameters of the immune response and inflammatory biomarkers, supporting recommendations to increase dietary fibers intake for enhanced immune health.

## Introduction

In recent decades, there has been a significant increase in abnormal inflammatory responses and immune system dysfunction, contributing to the development of chronic inflammatory disorders, as well as conditions such as cancer and diabetes (1–3). Therefore, the identification of potential regulators of inflammation and the immune system holds great significance in preventing and treating these diseases. It is well-established that changes in dietary factors play a crucial role in regulating immune function and inflammatory biomarkers (4). Both preclinical and clinical studies provide compelling evidence that a dietary shift from traditional diets abundant in plant-based foods to ultra-processed foods renders individuals susceptible to various chronic and debilitating inflammatory diseases (5, 6). Consequently, the influence of dietary nutrients on immune and inflammatory responses has emerged as an attractive and vital area of research. This study will specifically focus on one such dietary component: dietary fibers.

Dietary fibers are complex dietary components found mainly in grains, vegetables, and fruits that consist of three or more monomeric units (7, 8). These fibers are indigestible in the intestinal tract, but they play a unique and important role in the human body. Higher dietary fibers intake has been reported to improve immune responses and certain inflammatory disorders (8). *In vitro* and *in vivo* experiments have identified that dietary fibers impact immune cells through gut microbiota and may help prevent inflammatory conditions (9). More specifically, clinical studies suggest that dietary fibers act as protective factors against asthma (10), metabolic syndrome (11, 12), and radiation-induced gastrointestinal toxicity (13). Beyond diseases, a variety of immune and inflammatory biomarkers such as C-reactive protein, fibrinogen (14), tumor necrosis factor-α, and interleukin-10 (15) are associated with dietary fibers intake.

The systemic immune-inflammation index (SII) was first proposed by Hu et al. (16) as a prognostic predictor for hepatocellular carcinoma patients (16). However, the clinical interest in SII has grown significantly due to its ability to reflect systemic inflammation and immunity. Previous studies have established strong associations between SII and various diseases, including cancer (17), diabetes (18), hepatic steatosis (19), kidney injury (20), and cardiovascular risk (21). Similarly, the systemic inflammation response index (SIRI) was initially developed to predict the prognosis of pancreatic cancer, with higher levels of SIRI being linked to unfavorable prognostic outcomes (22). The neutrophil-to-lymphocyte ratio (NLR) and platelet-lymphocyte ratio (PLR) are calculated based on blood cell count and have been widely recognized as potential indicators for early diagnosis and prognosis monitoring in inflammatory diseases and cancers (23). Additionally, Red blood cell distribution width-to-albumin ratio (RA) has emerged as a novel inflammatory biomarker, showing associations with conditions such as stroke (24), diabetic ketoacidosis (25), acute respiratory distress syndrome (26), and all-cause mortality in cancer patients (27). Ferritin and high-sensitivity C-reactive protein (hs-CRP) are classical inflammatory biomarkers extensively used in routine clinical practice and inflammatory research.

Consequently, it has been established with certainty that these biomarkers can serve as reliable indicators of the immune and inflammatory condition of the human body, and they are correlated with various diseases that pose a threat to health. However, few studies have delved into whether these biomarkers can be modulated by dietary fibers. This study aimed to analyze the association between dietary fibers intake and systemic immunity and inflammation using data from the National Health and Nutrition Examination Survey (NHANES) survey conducted from 2015–2020.

## Materials and methods

### Study population

The NHANES is an epidemiological program developed to assess the health and nutritional conditions of adults and children in the United States. Conducted by the National Center for Health Statistics, a subdivision of the Centers for Disease Control and Prevention, NHANES combines interviews on demographic, socioeconomic, dietary, and health-related queries, physical examinations incorporating medical, dental, physiological measurements, and laboratory tests by highly qualified medical personnel. NHANES sample constitutes a representation of the noninstitutionalized civilian population in the United States, comprising the 50 states and the District of Columbia. From 1999 onwards, the sample design has employed a multi-year, stratified, clustered four-stage sampling approach, with data release in 2-year cycles.

**Figure 1:**
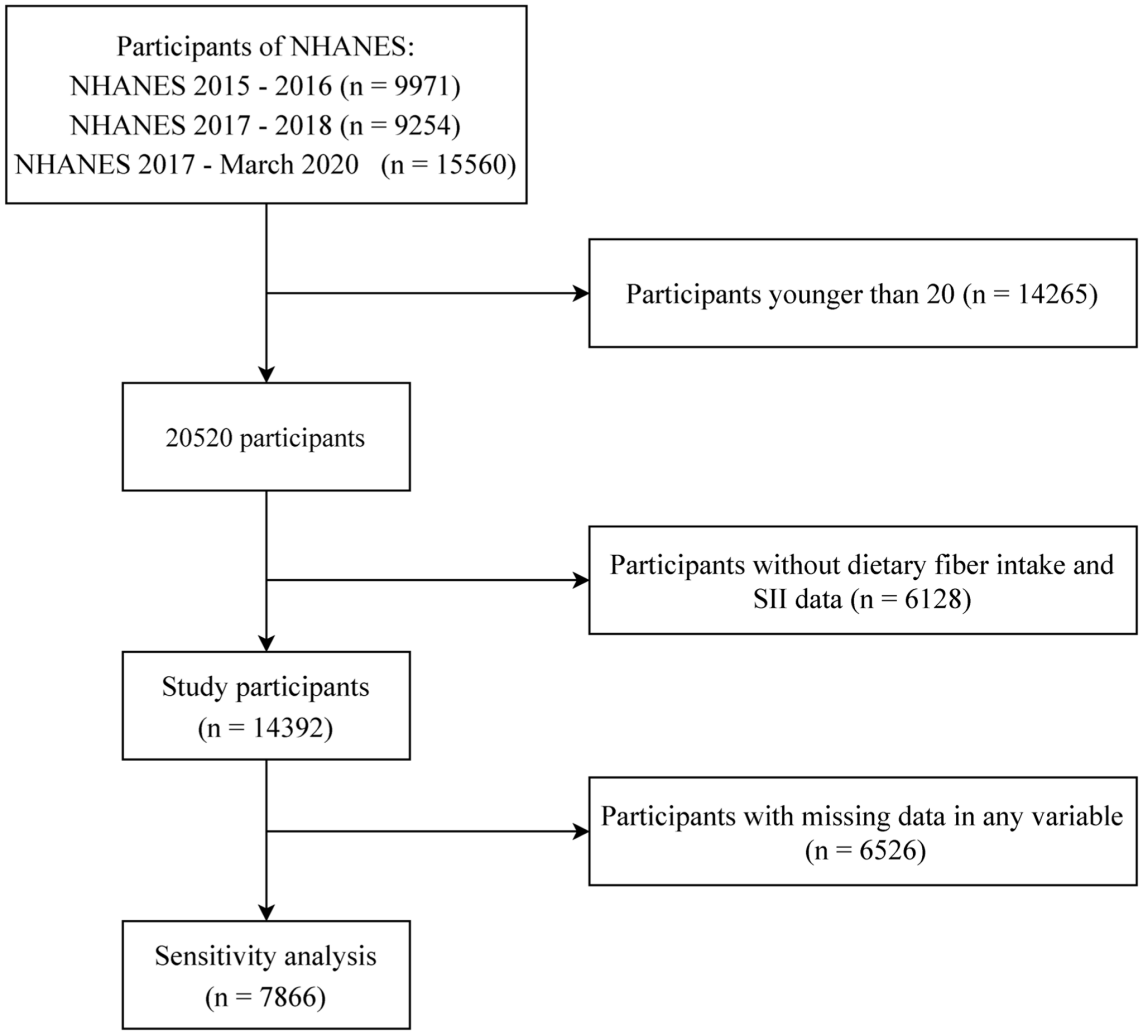
Participant screening flowchart based on age, dietary fibers intake, systemic immune/inflammatory biomarkers, and sensitivity analyses.

This study included NHANES data from 2015–2020. A total of 20,520 participants remained after excluding those younger than 20. We further excluded those lacking systemic immune-inflammation index (SII) or dietary fibers intake data, leaving 14,392 participants for the association analysis. In order to perform a sensitivity analysis with complete cases, 6,526 participants with incomplete data in any kind of variable were excluded. A flowchart presents the process of selecting participants (Figure 1).

### Measurement of dietary fibers intake

Dietary intake data was collected through two 24-h dietary recalls conducted 3–10 days apart during the Mobile Examination Center component of NHANES. The recalls were jointly processed by NHANES, the United States Department of Agriculture, and the United States Department of Health and Human Services. Average daily dietary fibers intake was calculated using the two 24-h of intake data. Full documentation of the dietary assessment methods is available in the NHANES dietary interviewer procedures manuals (28, 29).

### Measurement of primary and secondary outcomes

The primary outcome was the SII, calculated as: platelet counts × neutrophil count/lymphocyte count (16). SIRI, NLR, PLR, RA, ferritin, hs-CRP, and six kinds of white blood cell (WBC) count are the secondary outcomes of this study. The formulas for SIRI, NLR, PLR and RA are presented as follows: SIRI = neutrophil count × monocyte/lymphocyte count. (22), NLR = neutrophil counts/lymphocyte counts, PLR = platelet counts/lymphocyte counts, and RA = red blood cell distribution width (%)/albumin (mg/dl) (30). Ferritin and hs-CRP are well-acknowledged acute inflammation indicators obtained using blood specimen tests. NHANES provides standardized protocols for measuring these biomarkers, available on the NHANES website^1^ (31).

### Selection of covariates

Sociodemographic characteristics included age, gender (male and female), race (Mexican American, other Hispanic, non-Hispanic white, non-Hispanic black, and other), and family monthly poverty level index (≤1.3, 1.5–1.85, >1.85) were collected. Lifestyle characteristics included alcohol consumption (never, mild, moderate, and heavy), smoking status (never, former, and current), and vigorous recreational activity (Yes and No) were obtained. Never drinkers were ascertained by the questionnaire: “Ever had a drink of any kind of alcohol?” Furthermore, participants who had >4 drinks per day, 3–4 drinks per day, and up to 2 drinks per day were classified as heavy, moderate, and mild drinkers, respectively. Participants who smoked less than 100 cigarettes in life were considered as never smoking and the others were divided into former and current smokers according to the question “Do you now smoke cigarettes?” Metabolic characteristics included body mass index (BMI), hyperlipidemia, hypertension, and diabetes. An adult with a BMI below 18.5 kg/m2 is considered underweight, 18.5 to 24.9 is considered normal weight, 25 to 29.9 is considered overweight, and 30 or above is considered obesity. Hyperlipidemia was defined by high-density lipoprotein cholesterol <1.0 mmoL/L in men, < 1.3 mmoL/L in women, or triglycerides ≥1.8 mmoL/L regardless of gender. Hypertension was defined as systolic blood pressure ≥ 130 mmHg and/or diastolic blood pressure ≥ 80 mmHg on ≥3 occasions. Moreover, participants who take an anti-hypertensive agent or who answered “yes” to the questions: “Are you now taking prescribed medicine for high blood pressure?” and “Ever told you had high blood pressure?” were also defined as having hypertension. Diabetes was defined as a positive response to the question “Doctor told you have diabetes?.” Additionally, participants who achieved one or more of the following conditions were diagnosed with diabetes: glycohemoglobin ≥6.5%, fasting glucose ≥7 mmol/L, two-hour glucose of oral glucose tolerance test, or serum glucose ≥11.1 mmol/L. The dietary inflammatory index (DII) is a scoring algorithm developed through comprehensive analysis of scientific literature on the inflammatory properties of dietary components. The DII was used to categorize participants’ dietary patterns as either pro-inflammatory or anti-inflammatory (32).

### Statistical analyses

Dummy variables were used to denote missing covariate values. Continuous variables were presented as survey-weighted mean (95% confidence interval (CI)) and categorical variables were expressed as survey-weighted percentage (95% CI). The weighted χ2 test (categorical variable) and weighted linear regression model (continuous variable) compared tertiles of dietary fibers intake. A univariate and multivariate weighted linear regression model and/or weighted binary logistic regression model were used to examine the associations between dietary fibers intake and SII, as well as other outcomes. A total of three statistical models were constructed in each regression analysis. Model I was the non-adjusted model with no covariates adjusted. Model II was the minimally adjusted model with age and gender adjusted. Model III was a fully adjusted with age, gender, race, family monthly poverty level index, alcohol consumption, smoking status, vigorous recreational activities, BMI, hyperlipidemia, hypertension, diabetes and DII adjusted. The fully adjusted model took into account demographic factors, lifestyle factors, dietary factors, and metabolic factors. Covariates were selected by referring to cross-sectional studies related to our prespecified outcome indicators (33–36). Sensitivity analysis was conducted by excluding the participants with incomplete data in covariates. Taking the biochemical markers between human races into consideration (37), a subgroup analyses between races were performed using a stratified logistic regression model and a interaction test for effect modification for different races were followed by the likelihood-ratio test. Data analysis was performed with the statistical software packages R^2^ and EmpowerStats (http://www.empowerstats.com, X&Y Solutions, Inc., Boston, MA). All statistical tests were two-sided, and a *p* value <0.05 was considered statistically significant.

## Results

### Baseline characteristics

Table 1 shows baseline of the 14,392 participants by dietary fiber intake tertiles (low: 0–11.65 g/d, n = 4,790; middle: 11.7–18.45 g/d, n = 4,801; high: 18.5–89.55 g/d, *n* = 4,801). Participants with higher dietary fibers intake had lower levels of SII, SIRI, NLR, RA, hs-CRP, WBC, neutrophils and basophils. Furthermore, these individuals also exhibited exhibited a higher proportion of male and Mexican American participants, greater affluence, lower prevalence of obesity, and healthier lifestyle reflected by increased engagement in rigorous recreational activities, decreased usage of cigarettes and alcohol, and a higher percentage of adherence to an anti-inflammatory diet.

**Table 1:** Survey-weighted baseline characteristics by dietary fibers intake level in the study population.

Weighted variates		Low-DF (0–11.65 g/d) *N* = 4,790	Middle-DF (11.7–18.45 g/d) *N* = 4,801	High-DF (18.5–89.55 g/d) *N* = 4,801	Survey-weighted *p* value
SII [mean (95% CI), 1,000 cells/μL]		542.883 (527.361, 558.406)	526.415 (510.436, 542.394)	496.221 (482.761, 509.682)	<0.0001
SIRI [mean (95% CI), 1,000 cells/μL]		1.341 (1.299, 1.383)	1.320 (1.267, 1.373)	1.257 (1.206, 1.308)	0.03
NLR [mean (95% CI), ratio]		2.206 (2.155, 2.258)	2.161 (2.102, 2.220)	2.104 (2.049, 2.159)	0.012
PLR [mean (95% CI), ratio]		122.383 (120.215, 124.550)	120.189 (117.829, 122.548)	119.709 (117.444, 121.974)	0.208
RA [mean (95% CI), g%/dL]		3.367 (3.338, 3.396)	3.309 (3.281, 3.337)	3.212 (3.188, 3.235)	<0.0001
Ferritin [mean (95% CI), ug/L]		128.157 (122.460, 133.854)	141.200 (134.674, 147.726)	139.984 (127.380, 152.588)	0.007
hs-CRP [mean (95% CI), mg/L]		4.625 (4.201, 5.048)	4.128 (3.822, 4.434)	3.103 (2.801, 3.405)	<0.0001
WBC [mean (95% CI), 1,000 cells/μL]		7.639 (7.443, 7.835)	7.498 (7.341, 7.655)	7.159 (7.040, 7.279)	<0.0001
Neutrophils [mean (95% CI), 1,000 cells/μL]		4.471 (4.371, 4.571)	4.402 (4.290, 4.513)	4.153 (4.062, 4.245)	<0.0001
Lymphocyte [mean (95% CI), 1,000 cells/μL]		2.318 (2.181, 2.456)	2.247 (2.172, 2.322)	2.178 (2.130, 2.226)	0.063
Monocyte [mean (95% CI), 1,000 cells/μL]		0.596 (0.585, 0.607)	0.596 (0.584, 0.607)	0.584 (0.570, 0.597)	0.279
Eosinophils [mean (95% CI), 1,000 cells/μL]		0.204 (0.197, 0.211)	0.204 (0.195, 0.213)	0.197 (0.189, 0.205)	0.496
Basophils [mean (95% CI), 1,000 cells/μL]		0.059 (0.056, 0.062)	0.057 (0.054, 0.060)	0.052 (0.049, 0.055)	0.005
Age [mean (95% CI), years]		47.434 (46.436, 48.432)	49.106 (48.208, 50.004)	48.701 (47.677, 49.726)	0.008
Sex [percentage (95% CI)]					<0.0001
	male	39.157 (36.689, 41.683)	46.828 (44.678, 48.989)	56.959 (54.471, 59.413)	
	female	60.843 (58.317, 63.311)	53.172 (51.011, 55.322)	43.041 (40.587, 45.529)	
Race [percentage (95% CI)]					<0.0001
	Non-Hispanic White	62.833 (59.071, 66.446)	66.185 (61.848, 70.265)	62.027 (57.716, 66.156)	
	Mexican American	5.813 (4.629, 7.276)	8.206 (6.167, 10.842)	11.669 (9.226, 14.654)	
	Non-Hispanic Black	16.158 (13.702, 18.957)	10.854 (8.737, 13.409)	6.565 (5.312, 8.089)	
	Other Hispanic	6.751 (5.386, 8.431)	5.521 (4.359, 6.971)	8.267 (7.003, 9.735)	
	Other Race – Including Multi-Racial	8.445 (7.430, 9.583)	9.234 (7.834, 10.855)	11.472 (9.424, 13.897)	
FMMPLL [percentage (95% CI)]					<0.0001
	<= 1.3	26.278 (24.156, 28.515)	20.069 (17.950, 22.369)	17.362 (15.396, 19.521)	
	>1.3, <= 1.85	13.273 (11.677, 15.050)	11.045 (9.634, 12.633)	10.105 (8.987, 11.344)	
	>1.85	53.820 (51.438, 56.184)	62.243 (59.207, 65.186)	66.511 (64.082, 68.855)	
	Not obtained	6.630 (5.603, 7.829)	6.644 (5.434, 8.099)	6.022 (4.914, 7.362)	
Diabetes [percentage (95% CI)]					0.171
	NO	83.600 (81.727, 85.316)	82.065 (80.292, 83.711)	83.725 (82.097, 85.232)	
	YES	15.744 (14.036, 17.618)	16.905 (15.313, 18.627)	15.081 (13.641, 16.644)	
	NA	0.655 (0.422, 1.016)	1.030 (0.634, 1.669)	1.193 (0.865, 1.644)	
Hyperlipidemia [percentage (95% CI)]					0.586
	NO	31.425 (29.068, 33.881)	30.583 (28.185, 33.092)	33.367 (30.609, 36.244)	
	YES	68.575 (66.119, 70.932)	69.415 (66.907, 71.814)	66.633 (63.756, 69.391)	
	NA	0.000 (0.000, 0.000)	0.001 (0.000, 0.009)	0.000 (0.000, 0.000)	
Hypertension [percentage (95% CI)]					0.458
	NO	63.988 (61.280, 66.610)	62.096 (59.801, 64.339)	63.497 (61.038, 65.887)	
	YES	36.012 (33.390, 38.720)	37.904 (35.661, 40.199)	36.503 (34.113, 38.962)	
BMI level [percentage (95% CI)]					0.004
	Not obtained	0.486 (0.337, 0.699)	0.630 (0.446, 0.889)	0.562 (0.316, 0.999)	
	< 18.5	1.527 (1.003, 2.320)	1.285 (0.882, 1.870)	0.851 (0.585, 1.235)	
	> = 18.5, <= 24.9	23.590 (21.320, 26.022)	23.686 (21.233, 26.328)	26.292 (23.733, 29.022)	
	> = 25, <= 29.9	28.935 (26.200, 31.832)	31.595 (29.270, 34.015)	33.729 (31.111, 36.452)	
	> = 30	45.462 (42.885, 48.064)	42.804 (40.205, 45.443)	38.565 (35.530, 41.692)	
VRA [percentage (95% CI)]					<0.0001
	NO	76.953 (74.506, 79.230)	72.588 (70.399, 74.673)	61.764 (58.315, 65.099)	
	YES	23.047 (20.770, 25.494)	27.412 (25.327, 29.601)	38.236 (34.901, 41.685)	
Smoking status [percentage (95% CI)]					<0.0001
	NA	0.045 (0.012, 0.172)	0.024 (0.003, 0.173)	0.077 (0.011, 0.511)	
	never	51.986 (48.822, 55.135)	59.600 (56.379, 62.741)	61.553 (59.628, 63.443)	
	former	23.339 (21.417, 25.378)	25.067 (22.860, 27.411)	27.631 (25.813, 29.526)	
	current	24.630 (22.153, 27.286)	15.309 (13.453, 17.370)	10.739 (9.395, 12.250)	
Alcohol consumption [percentage (95% CI)]					0.023
	NA	14.551 (12.761, 16.544)	12.547 (11.446, 13.737)	12.228 (10.711, 13.925)	
	never	7.957 (6.975, 9.064)	8.430 (7.058, 10.040)	9.283 (7.841, 10.959)	
	former	4.334 (3.496, 5.360)	4.792 (3.674, 6.228)	4.379 (3.515, 5.443)	
	midl	47.414 (44.605, 50.240)	51.943 (49.015, 54.858)	49.138 (45.656, 52.629)	
	moderate	16.076 (13.938, 18.471)	15.984 (13.996, 18.195)	15.503 (13.581, 17.641)	
	heavy	9.668 (8.280, 11.260)	6.304 (5.037, 7.864)	9.469 (8.024, 11.143)	
DII [percentage (95% CI)]					<0.0001
	Pro-inflammatory diet	96.616 (95.686, 97.351)	84.194 (82.127, 86.063)	49.016 (46.087, 51.952)	
	Anti-inflammatory diet	3.384 (2.649, 4.314)	15.806 (13.937, 17.873)	50.984 (48.048, 53.913)	

SII, systemic immune-inflammation index; SIRI, systemic inflammation response index; NLR, neutrophil-to-lymphocyte ratio; PLR, platelet-lymphocyte ratio; RA, red blood cell distribution width-to-albumin ratio; hs-CRP, high-sensitivity C-Reactive Protein; WBC, white blood cell; FMMPLL, family monthly poverty level index; VRA, vigorous recreational activities; BMI, body mass index, DII, dietary inflammatory index, CI, confidence interval, DF, dietary fiber.

### Associations between dietary fibers intake and SII, SIRI, NLR, and PLR

Dietary fibers intake shows significant inverse associations with SII, SIRI, NLR in all 3 models (Table 2). The effect size (β) and 95% confidence interval (CI) for SII in the fully-adjusted model are −2.19885 (−3.21476, −1.18294) and the highest tertile significantly associated with decreased SII (β = −43.29833, 95% CI: −67.46845 to −19.12821, *p* = 0.001073). The *p* for trend across dietary fibers intake categories reaches statistical significance (β_trend_ = −21.5411, 95% CI: −33.0049 to −10.0772, *P_trend_* = 0.0011).

**Table 2:** Survey-weighted univariate and multivariate regression analyses of associations between dietary fibers intake and SII, SIRI, NLR, and PLR.

Exposure	Non-adjusted model, β (95%CI) P	Minimally-adjusted model, β (95%CI) P	Fully-adjusted model, β (95%CI) P
SII			
Dietary fiber	−2.48688 (−3.32054, −1.65322) <0.000001	−2.21499 (−3.03924, −1.39074) 0.000003	−2.19885 (−3.21476, −1.18294) 0.000248
Dietary fiber tertiles			
Low	Ref	Ref	Ref
Middle	−16.46863 (−35.98249, 3.04523) 0.104012	−15.81623 (−34.70707, 3.07462) 0.106952	−16.55926 (−36.44972, 3.3312) 0.098115
High	−46.66205 (−65.77292, −27.55118) 0.000014	−42.19161 (−60.33388, −24.04934) 0.000033	−43.29833 (−67.46845, −19.12821) 0.001073
P trend	−23.4625 (−32.9424, −13.9825) <0.0001	−21.1946 (−30.2038, −12.1854) <0.0001	−21.5411 (−33.0049, −10.0772) 0.0011
SIRI			
Dietary fiber	−0.00474 (−0.00753, −0.00195) 0.001555	−0.00713 (−0.00997, −0.0043) 0.000009	−0.00642 (−0.01021, −0.00263) 0.001738
Dietary fiber tertiles			
Low	Ref	Ref	Ref
Middle	−0.02079 (−0.08507, 0.04349) 0.528897	−0.05021 (−0.11587, 0.01545) 0.140109	−0.04873 (−0.11394, 0.01648) 0.13556
High	−0.0838 (−0.14515, −0.02245) 0.009862	−0.13215 (−0.19208, −0.07221) 0.000072	−0.12477 (−0.20495, −0.04459) 0.003611
P trend	−0.0423 (−0.0730, −0.0116) 0.0093	−0.0664 (−0.0964, −0.0364) 0.0001	−0.0621 (−0.1000, −0.0242) 0.0035
NLR			
Dietary fiber	−0.00448 (−0.00757, −0.00139) 0.006316	−0.00601 (−0.00903, −0.00299) 0.000278	−0.00803 (−0.01179, −0.00427) 0.000284
Dietary fiber tertiles			
Low	Ref	Ref	Ref
Middle	−0.0451 (−0.11806, 0.02786) 0.231031	−0.07218 (−0.14502, 0.00065) 0.057609	−0.09348 (−0.16845, −0.0185) 0.016393
High	−0.1024 (−0.16757, −0.03723) 0.003281	−0.13784 (−0.19768, −0.078) 0.000038	−0.18596 (−0.26639, −0.10553) 0.000067
P trend	−0.0513 (−0.0839, −0.0187) 0.0032	−0.0689 (−0.0988, −0.0389) <0.0001	−0.0930 (−0.1312, −0.0547) 0.0001
PLR			
Dietary fiber	−0.0566 (−0.19744, 0.08424) 0.434314	−0.00215 (−0.13867, 0.13436) 0.975435	−0.13014 (−0.29189, 0.03161) 0.126899
Dietary fiber tertiles			
Low	Ref	Ref	Ref
Middle	−2.19396 (−5.08296, 0.69503) 0.142554	−2.17535 (−4.98881, 0.6381) 0.135829	−3.11979 (−5.74119, −0.4984) 0.028014
High	−2.6734 (−5.90449, 0.55769) 0.110802	−1.91749 (−5.12276, 1.28778) 0.246431	−4.48801 (−7.92369, −1.05234) 0.016881
P trend	−1.3203 (−2.9431, 0.3025) 0.1166	−0.9360 (−2.5470, 0.6751) 0.2600	−2.2626 (−3.9648, −0.5604) 0.0150

SII, systemic immune-inflammation index; SIRI, systemic inflammation response index; NLR, neutrophil-to-lymphocyte ratio; PLR, platelet-lymphocyte ratio; OR, odds ratio; CI, confidence interval.

Non-adjusted model: no covariates were adjusted. Minimally-adjusted model: age and gender were adjusted. Fully-adjusted model: age, gender, race, family monthly poverty level index, alcohol consumption, smoking status, vigorous recreational activities, body mass index level, hyperlipidemia, hypertension, diabetes, and dietary inflammatory index.

The β and 95% CI for SIRI in the fully-adjusted model are −0.00642 (−0.01021, −0.00263) and the highest tertile significantly associated with decreased SIRI (β = −0.12477, 95% CI: −0.20495 to −0.04459, *p* = 0.003611). The *p* for trend across dietary fibers intake categories reaches statistical significance (β_trend_ = −0.0621, 95% CI: −0.1000 to −0.0242, *P_trend_* = 0.0035).

The β and 95% CI for NLR in the fully-adjusted model are −0.00803 (−0.01179, −0.00427) and the highest tertile significantly associated with decreased NLR (β = −0.18596, 95% CI: −0.26639 to −0.10553, *p* = 0.000067). The *p* for trend across dietary fibers intake categories reaches statistical significance (β_trend_ = −0.0930, 95% CI: −0.1312 to −0.0547, *P_trend_* = 0.0001).

The β and 95% CI for PLR in the fully-adjusted model are −0.13014 (−0.29189, 0.03161) and the highest tertile significantly associated with decreased PLR (β = −4.48801, 95% CI: −7.92369 to −1.05234, *p =* 0.016881). The *P* for trend across dietary fibers intake categories reaches statistical significance (β_trend_ = −2.2626, 95% CI: −3.9648 to −0.5604, *P_trend =_* 0.0150).

### Associations between dietary fibers intake and RA

Dietary fibers intake presents significant inverse associations with RA (β = −0.00266, 95% CI: −0.00401 to −0.00131, *p* = 0.000644). The β and 95% CI for the highest tertile is −0.07064 (−0.010227, −0.03901) in the fully-adjusted model. A significant negative trend is observed across dietary fiber intake categories (β_trend_ = −0.0351, 95% CI: −0.0503 to −0.0199, *P_trend_* = 0.0001). The β and corresponding 95% CI for all the statistical models are presented in Table 3.

**Table 3:** Survey-weighted univariate and multivariate regression analyses of the association between dietary fibers intake and RA.

Exposure	Non-adjusted model, β (95%CI) P	Minimally-adjusted model, β (95%CI) P	Fully-adjusted model, β (95%CI) P
RA			
Dietary fiber	−0.00744 (−0.00903, −0.00586) <0.000001	−0.00576 (−0.00734, −0.00418) <0.000001	−0.00266 (−0.00401, −0.00131) 0.000644
Dietary fiber tertiles			
Low	Ref	Ref	Ref
Middle	−0.05793 (−0.08976, −0.02611) 0.000773	−0.05149 (−0.08072, −0.02227) 0.001122	−0.02287 (−0.05325, 0.00751) 0.133181
High	−0.15514 (−0.1925, −0.11778) <0.000001	−0.12805 (−0.16465, −0.09145) <0.000001	−0.07064 (−0.10227, −0.03901) 0.000097
P trend	−0.0780 (−0.0968, −0.0591) <0.0001	−0.0643 (−0.0827, −0.0459) <0.0001	−0.0351 (−0.0503, −0.0199) 0.0001

RA, red blood cell distribution width-to-albumin ratio.

Non-adjusted model: no covariates were adjusted. Minimally-adjusted model: age and gender were adjusted. Fully-adjusted model: age, gender, race, family monthly poverty level index, alcohol consumption, smoking status, vigorous recreational activities, body mass index level, hyperlipidemia, hypertension, diabetes, and dietary inflammatory index.

### Associations between dietary fibers intake and ferritin and hs-CRP

An inverse association is observed between dietary fibers intake and ferritin (β = −0.73086, 95% CI: −1.31385 to −0.14787, *p* = 0.020716). However, when we stratified dietary fibers into tertiles, statistical significance was not attained in any tertile or across tertiles (Table 4). Dietary fibers intake also shows an inverse correlation with hs-CRP (β = −0.04629, 95% CI: −0.0743 to −0.01829, *p* = 0.002119), with the highest tertile significantly associated with decreased hs-CRP (β = −0.8598, 95% CI: −1.49918 to −0.22043, *p =* 0.010218) and the *P* for trend across dietary fibers intake categories reaches statistical significance (β_trend_ = −0.4261, 95% CI: −0.7299 to −0.1224, *P_trend =_* 0.0105).

**Table 4:** Survey-weighted univariate and multivariate regression analyses of the associations between dietary fibers intake and ferritin and hs-CRP.

Exposure	Non-adjusted model, β (95%CI) P	Minimally-adjusted model, β (95%CI) P	Fully-adjusted model, β (95%CI) P
Ferritin			
Dietary fiber	0.29962 (−0.26961, 0.86886) 0.306821	−0.85335 (−1.32295, −0.38376) 0.000798	−0.73086 (−1.31385, −0.14787) 0.020716
Dietary fiber tertiles			
Low	Ref	Ref	Ref
Middle	13.04284 (4.70996, 21.37572) 0.003393	0.92668 (−6.62744, 8.4808) 0.810956	3.11558 (−3.64433, 9.87549) 0.374636
High	11.82705 (−2.60333, 26.25744) 0.114127	−9.73583 (−20.30168, 0.83002) 0.076817	−5.24483 (−18.5334, 8.04373) 0.446152
P trend	5.8988 (−1.3422, 13.1397) 0.1162	−4.8841 (−10.1972, 0.4290) 0.0774	−2.3954 (−8.8288, 4.0381) 0.4718
hs-CRP			
Dietary fiber	−0.08331 (−0.10509, −0.06152) <0.000001	−0.07581 (−0.09749, −0.05413) <0.000001	−0.04629 (−0.0743, −0.01829) 0.002119
Dietary fiber tertiles			
Low	Ref	Ref	Ref
Middle	−0.49671 (−0.96947, −0.02395) 0.044393	−0.44696 (−0.91731, 0.0234) 0.068297	−0.23148 (−0.70177, 0.23881) 0.320052
High	−1.52145 (−2.04615, −0.99675) <0.000001	−1.38093 (−1.90503, −0.85683) 0.000004	−0.8598 (−1.49918, −0.22043) 0.010218
P trend	−0.7661 (−1.0272, −0.5049) <0.0001	−0.6954 (−0.9563, −0.4345) <0.0001	−0.4261 (−0.7299, −0.1224) 0.0105

hs-CRP, high-sensitivity C-Reactive Protein; OR, odds ratio; CI, confidence interval.

Non-adjusted model: no covariates were adjusted. Minimally-adjusted model: age and gender were adjusted. Fully-adjusted model: age, gender, race, family monthly poverty level index, alcohol consumption, smoking status, vigorous recreational activities, body mass index level, hyperlipidemia, hypertension, diabetes, and dietary inflammatory index.

### Associations between dietary fibers intake and white blood cell

Significant inverse correlations are observed between dietary fibers intake and WBC (β = −0.01624, 95% CI: −0.02685 to −0.00563, *p* = 0.004066; β_trend_ = −0.1268, 95% CI: −0.2277 to −0.0258, *P_trend_* = 0.0209), and neutrophils (β = −0.01346, 95% CI: −0.01929 to −0.00764, *p* < 0.000064; β_trend_ = −0.1047, 95% CI: −0.1641 to −0.0453, *P_trend_* = 0.0019) (Table 5). However, no significant associations are seen with lymphocytes, monocytes, eosinophils or basophils.

**Table 5:** Survey-weighted univariate and multivariate regression analyses of the association between dietary fibers intake and WBC.

Exposure	Non-adjusted model, β (95%CI) P	Minimally-adjusted model, β (95%CI) P	Fully-adjusted model, β (95%CI) P
WBC			
Dietary fiber	−0.02843 (−0.0357, −0.02116) <0.000001	−0.02688 (−0.03436, −0.0194) <0.000001	−0.01624 (−0.02685, −0.00563) 0.004066
Dietary fiber tertiles			
Low	Ref	Ref	Ref
Middle	−0.14077 (−0.37434, 0.0928) 0.242777	−0.11933 (−0.35091, 0.11225) 0.31729	−0.04567 (−0.24459, 0.15325) 0.656584
High	−0.4794 (−0.67069, −0.28811) 0.000009	−0.442 (−0.63081, −0.25319) 0.000029	−0.25703 (−0.46003, −0.05402) 0.02016
P trend	−0.2416 (−0.3357, −0.1475) <0.0001	−0.2229 (−0.3158, −0.1300) <0.0001	−0.1268 (−0.2277, −0.0258) 0.0209
Neutrophils
Dietary fiber	−0.01934 (−0.02451, −0.01418) <0.000001	−0.01832 (−0.02361, −0.01303) <0.000001	−0.01346 (−0.01929, −0.00764) 0.000064
Dietary fiber tertiles			
Low	Ref	Ref	Ref
Middle	−0.06914 (−0.20712, 0.06883) 0.33046	−0.05651 (−0.19298, 0.07996) 0.420792	−0.02977 (−0.15053, 0.09098) 0.633115
High	−0.31726 (−0.42947, −0.20506) <0.000001	−0.29314 (−0.40377, −0.18251) 0.000004	−0.21267 (−0.33229, −0.09305) 0.001835
P trend	−0.1603 (−0.2158, −0.1049) <0.0001	−0.1483 (−0.2030, −0.0935) <0.0001	−0.1047 (−0.1641, −0.0453) 0.0019
Lymphocyte			
Dietary fiber	−0.0074 (−0.01113, −0.00367) 0.00028	−0.0059 (−0.00947, −0.00234) 0.002064	−0.00183 (−0.00863, 0.00497) 0.58431
Dietary fiber tertiles			
Low	Ref	Ref	Ref
Middle	−0.07136 (−0.22374, 0.08101) 0.362818	−0.05303 (−0.20219, 0.09613) 0.489059	−0.01814 (−0.1611, 0.12483) 0.795617
High	−0.14025 (−0.28676, 0.00625) 0.06612	−0.10871 (−0.24628, 0.02885) 0.127573	−0.03531 (−0.18774, 0.11712) 0.636733
P trend	−0.0701 (−0.1422, 0.0020) 0.0620	−0.0544 (−0.1221, 0.0133) 0.1214	−0.0177 (−0.0903, 0.0550) 0.6376
Monocyte			
Dietary fiber	−0.00108 (−0.00173, −0.00042) 0.002248	−0.00173 (−0.00242, −0.00104) 0.000009	−0.00093 (−0.00188, 0.00003) 0.056673
Dietary fiber tertiles			
Low	Ref	Ref	Ref
Middle	−0.00065 (−0.01447, 0.01316) 0.926339	−0.00676 (−0.02073, 0.0072) 0.346969	−0.00069 (−0.01311, 0.01172) 0.913595
High	−0.0124 (−0.02897, 0.00417) 0.148336	−0.02452 (−0.04134, −0.0077) 0.006171	−0.00926 (−0.02949, 0.01096) 0.377849
P trend	−0.0063 (−0.0146, 0.0020) 0.1439	−0.0124 (−0.0208, −0.0039) 0.0060	−0.0045 (−0.0146, 0.0055) 0.3817
Eosinophils			
Dietary fiber	−0.00039 (−0.00089, 0.0001) 0.121687	−0.00067 (−0.0012, −0.00015) 0.015107	0.00008 (−0.00052, 0.00067) 0.802277
Dietary fiber tertiles			
Low	Ref	Ref	Ref
Middle	−0.00043 (−0.0093, 0.00843) 0.923931	−0.00328 (−0.01214, 0.00557) 0.470761	0.00131 (−0.00756, 0.01019) 0.7741
High	−0.00699 (−0.01861, 0.00463) 0.243849	−0.01232 (−0.02431, −0.00033) 0.049358	0.00017 (−0.01265, 0.01299) 0.979598
P trend	−0.0036 (−0.0094, 0.0023) 0.2422	−0.0062 (−0.0123, −0.0001) 0.0501	0.0001 (−0.0062, 0.0064) 0.9729
Basophils			
Dietary fiber	−0.00031 (−0.00045, −0.00016) 0.000122	−0.0003 (−0.00045, −0.00016) 0.000155	−0.00009 (−0.00029, 0.0001) 0.359748
Dietary fiber tertiles			
Low	Ref	Ref	Ref
Middle	−0.00232 (−0.00581, 0.00118) 0.199226	−0.0027 (−0.00613, 0.00073) 0.129011	−0.00089 (−0.00449, 0.00272) 0.633727
High	−0.00668 (−0.01058, −0.00279) 0.001442	−0.00683 (−0.0107, −0.00297) 0.001089	−0.00347 (−0.00797, 0.00102) 0.142744
P trend	−0.0034 (−0.0053, −0.0014) 0.0014	−0.0034 (−0.0054, −0.0015) 0.0010	−0.0017 (−0.0040, 0.0005) 0.1449

WBC, white blood cell; OR, odds ratio; CI, confidence interval.

Non-adjusted model: no covariates were adjusted. Minimally-adjusted model: age and gender were adjusted. Fully-adjusted model: age, gender, race, family monthly poverty level index, alcohol consumption, smoking status, vigorous recreational activities, body mass index level, hyperlipidemia, hypertension, diabetes, and dietary inflammatory index.

### Sensitivity analysis of complete cases for SII

Sensitivity analysis continues to show an inverse association between dietary fibers intake and SII (Table 6). The β and 95% CI are −2.0099 (−3.08293, −0.93687) for the non-adjusted model, −1.7928 (−2.85182, −0.73377) for the minimally-adjusted model, and − 1.59067 (−3.09644, −0.08491) for fully-adjusted model. The highest tertile (β = −34.10908, 95% CI: −65.05815 to −3.16001, *p* = 0.03861) is significantly associated with decreased SII in fully-adjusted model. The *P* for trend across dietary fibers intake categories reaches statistical significance (β_trend_ = −17.2185, 95% CI: −32.7649 to −1.6721, *P_trend_* = 0.0375). Additionally, robust inverse associations are also observed between dietary fibers intake and SIRI, NLR, RA, ferritin, hs-CRP, WBC, and neutrophils (Supplementary Table S1).

**Table 6:** Sensitivity analysis for the association between dietary fibers intake and SII.

Exposure	Non-adjusted model, β (95%CI) P	Minimally-adjusted model, β (95%CI) P	Fully-adjusted model, β (95%CI) P
SII
Dietary fiber	−2.0099 (−3.08293, −0.93687) 0.000554	−1.7928 (−2.85182, −0.73377) 0.00166	−1.59067 (−3.09644, −0.08491) 0.038831
Dietary fiber tertiles
Low	Ref	Ref	Ref
Middle	−26.80641 (−50.98476, −2.62806) 0.034268	−25.94692 (−49.43197, −2.46188) 0.035053	−23.67319 (−48.36226, 1.01589) 0.069626
High	−38.6293 (−64.27285, −12.98576) 0.004689	−36.1454 (−60.8432, −11.4476) 0.005986	−34.10908 (−65.05815, −3.16001) 0.03861
P trend	−19.1824 (−31.9955, −6.3693) 0.0049	−17.9224 (−30.2670, −5.5778) 0.0063	−17.2185 (−32.7649, −1.6721) 0.0375

SII, systemic immune-inflammation index; OR, odds ratio; CI, confidence interval.

Non-adjusted model: no covariates were adjusted. Minimally-adjusted model: age and gender were adjusted. Fully-adjusted model: age, gender, race, family monthly poverty level index, alcohol consumption, smoking status, vigorous recreational activities, body mass index level, hyperlipidemia, hypertension, diabetes, and dietary inflammatory index.

### Interaction effect of race on the associations between dietary fibers intake and outcomes

Interaction tests showed no significant difference in the associations between dietary fibers intake and systemic immune and inflammatory biomarkers by race (Table 7). The *P_interaction_* for race and SII, SIRI, NLR, PLR, RA, ferritin, hs-CRP, and six kinds of WBC count were 0.9941, 0.9085, 0.9054, 0.0495, 0.6856, 0.476, 0.1873, 0.3227, 0.1548, 0.2794, 0.2081, 0.659 and 0.6209.

**Table 7:** Associations between dietary fibers and outcomes in different races.

Outcome	Non-Hispanic white	Mexican american	Non-hispanic black	Other hispanic	Other race – including multi-racial	Survey-weighted P interaction
SII	−2.1584 (−3.5905, −0.7263) 0.0073	−2.1199 (−3.6506, −0.5891) 0.0127	−2.0635 (−3.5913, −0.5356) 0.0147	−2.1489 (−3.6529, −0.6448) 0.0104	−2.5560 (−4.3696, −0.7424) 0.0114	0.9941
SIRI	−0.0059 (−0.0111, −0.0008) 0.0350	−0.0059 (−0.0114, −0.0004) 0.0463	−0.0083 (−0.0143, −0.0023) 0.0123	−0.0073 (−0.0111, −0.0036) 0.0009	−0.0074 (−0.0121, −0.0028) 0.0046	0.9085
NLR	−0.0078 (−0.0135, −0.0020) 0.0143	−0.0082 (−0.0145, −0.0019) 0.0185	−0.0091 (−0.0144, −0.0038) 0.0028	−0.0067 (−0.0122, −0.0012) 0.0265	−0.0093 (−0.0144, −0.0043) 0.0015	0.9054
PLR	−0.0117 (−0.2605, 0.2371) 0.9275	−0.2484 (−0.4497, −0.0472) 0.0242	−0.3982 (−0.6300, −0.1663) 0.0028	−0.2636 (−0.5567, 0.0295) 0.0918	−0.3085 (−0.5458, −0.0712) 0.0183	0.0495
RA	−0.0027 (−0.0042, −0.0011) 0.0024	−0.0032 (−0.0060, −0.0003) 0.0381	−0.0040 (−0.0073, −0.0006) 0.0296	−0.0034 (−0.0055, −0.0013) 0.0048	−0.0008 (−0.0047, 0.0030) 0.6714	0.6856
Ferritin	−0.9309 (−1.5520, −0.3098) 0.0074	−0.5142 (−1.5663, 0.5379) 0.3481	0.0393 (−0.7400, 0.8185) 0.9222	−0.8746 (−2.0068, 0.2575) 0.1436	−0.5149 (−2.1876, 1.1579) 0.5522	0.476
hs-CRP	−0.0484 (−0.0839, −0.0129) 0.0137	−0.0734 (−0.1036, −0.0431) 0.0001	−0.0278 (−0.0698, 0.0142) 0.2072	−0.0294 (−0.0679, 0.0091) 0.1483	−0.0350 (−0.0762, 0.0063) 0.1106	0.1873
WBC	−0.0213 (−0.0346, −0.0080) 0.0047	−0.0072 (−0.0214, 0.0069) 0.3261	−0.0149 (−0.0303, 0.0004) 0.0696	−0.0064 (−0.0197, 0.0068) 0.3535	−0.0088 (−0.0231, 0.0054) 0.2355	0.3227
Neutrophils	−0.0169 (−0.0244, −0.0093) 0.0002	−0.0085 (−0.0197, 0.0028) 0.1539	−0.0048 (−0.0143, 0.0047) 0.3326	−0.0088 (−0.0178, 0.0001) 0.0664	−0.0107 (−0.0198, −0.0017) 0.0299	0.1548
Lymphocyte	−0.0033 (−0.0116, 0.0050) 0.4405	0.0017 (−0.0049, 0.0082) 0.6217	−0.0086 (−0.0241, 0.0070) 0.2917	0.0040 (−0.0033, 0.0113) 0.2923	0.0018 (−0.0041, 0.0077) 0.5622	0.2794
Monocyte	−0.0008 (−0.0021, 0.0004) 0.1913	−0.0005 (−0.0019, 0.0008) 0.4331	−0.0015 (−0.0026, −0.0003) 0.0231	−0.0017 (−0.0028, −0.0006) 0.0058	−0.0008 (−0.0017, 0.0001) 0.0887	0.2081
Eosinophils	−0.0001 (−0.0008, 0.0007) 0.8063	−0.0000 (−0.0007, 0.0006) 0.8913	−0.0000 (−0.0009, 0.0009) 0.9955	−0.0001 (−0.0026, 0.0024) 0.9360	0.0011 (−0.0004, 0.0026) 0.1590	0.659
Basophils	−0.0001 (−0.0003, 0.0002) 0.6171	−0.0002 (−0.0005, 0.0002) 0.3254	0.0001 (−0.0003, 0.0004) 0.7134	−0.0003 (−0.0007, 0.0000) 0.0945	−0.0001 (−0.0004, 0.0003) 0.6843	0.6209

SII, systemic immune-inflammation index; SIRI, systemic inflammation response index; NLR, neutrophil-to-lymphocyte ratio; PLR, platelet-lymphocyte ratio; RA, red blood cell distribution width-to-albumin ratio; hs-CRP, high-sensitivity C-Reactive Protein; WBC, white blood cell.

Aage, gender, family monthly poverty level index, alcohol consumption, smoking status, vigorous recreational activities, body mass index level, hyperlipidemia, hypertension, diabetes, and dietary inflammatory index were adjusted.

## Discussion

This study conducted a comprehensive cross-sectional investigation using data from the NHANES 2015–2020 survey, which represents the U.S. population, to explore the association between dietary fibers intake and systemic immune and inflammatory biomarkers. The results of our study indicate that dietary fibers intake is inversely associated with SII, SIRI, NLR, RA, hs-CRP, WBC, and neutrophils. Furthermore, the sensitivity analysis confirmed the robustness of these findings. To the best of our knowledge, this is the initial investigation to evaluate such associations within a nationally representative sample.

SII, SIRI, NLR, and RA are potent biomarkers of the body’s immune and inflammatory state and have demonstrated predictive value for a wide range of diseases. The role of dietary factors as potential regulators of these biomarkers is evident in the literature review. In a case–control study involving 527 participants, dietary inflammation levels in women with polycystic ovary syndrome showed a positive correlation with SII, NLR, and PLR (38). Similarly, in a cross-sectional study with 1,050 participant, dietary inflammation level was positively associated with SIRI in individuals with mild cognitive impairment (39). Another study revealed a negative correlation between dietary antioxidant capacity and NLR in cancer patients (40). Additionally, a retrospective study found that dietary omega- 6 to omega- 3 fatty acids was associated with reduced PLR level in men with chronic coronary syndrome. (41). Our study unveiled an inverse association between dietary fibers and SII, SIRI, NLR, and RA, suggesting that a high-fiber diet may help regulate these biomarkers and potentially benefit the immune system.

Ferritin, initially identified as a reactant of acute inflammation caused by infectious agents, has subsequently been linked to acute and chronic inflammatory conditions precipitated by non-infectious sources. Moreover, it has been been demonstrated to play a pivotal role in the pathogenesis of various inflammatory and autoimmune diseases (42, 43). The rapid elevation in serum ferritin levels at the onset of viral or bacterial infections renders it a sensitive biomarker with clinical utility (44). However, it takes up to 5 weeks for ferritin levels to decrease (45). Elevated ferritin levels have been shown to be associated with autoimmune diseases such as rheumatoid arthritis, systemic lupus erythematosus, and multiple sclerosis, in which ferritin predicts disease severity or contributes to disease development (46–48). *In vitro* experiments have shown that Low Phytate Peas containing dietary fibers can affect hepatic ferritin concentrations (49). However, the relationship between dietary fibers intake and ferritin is controversial in clinical and cross-sectional studies. A prospective, randomized, placebo-controlled clinical trial conducted in China on end-stage renal disease patients treated with dietary fibers (composed of galactomannan, resistant dextrin, fructooligosaccharide, and starch) or potato starch for 8 weeks showed that the patients in the dietary fibers group had higher serum ferritin levels (50). Another randomized, double-blind, placebo-controlled with 32 female athletes demonstrated that daily synbiotic supplement along with Fe supplementation increased serum ferritin levels (51). However, some studies have come to the opposite conclusion. In a crossover-design clinical trial, healthy participants who took a high-fiber snack for 6 weeks and maintained it with a low-fiber snack for 6 weeks had lower ferritin levels compared to the control group (52). A French epidemiological survey study that included 4,358 subjects also found a negative association between dietary fibers intake and serum ferritin levels (53). In addition, vegans with high-fiber diets have been found to have low ferritin levels in several dietary investigations (54). Although the fully adjusted model indicated that dietary fibers intake was negatively associated with ferritin levels, there was no statistical difference in the analysis of the trend test, making the relationship between dietary fibers intake and ferritin unstable in our study. Hs-CRP is a biomarker of systemic inflammation in the body, in addition to being regarded as an indicator of acute inflammation, it is associated with many chronic diseases, including coronary heart disease (55), metabolic syndrome (56), diabetes mellitus (57), and cancer (58). Evidence of an inverse correlation between the dietary fibers intake and hs-CRP concentrations has emerged from multiple cohort studies conducted on the American population. Two cross-sectional analyses of NHANES data from 1999–2000 included 3,920 and 4,900 participants, respectively (59, 60). Concurrently, a longitudinal cohort study involving 524 healthy adults (61) and a small clinical trial have been conducted (62). A parallel dietary intervention trial has demonstrated that incorporating high-fiber wholegrain rye foods with added fermented rye bran led to a reduction in hs-CRP levels among Chinese adults (63). However, no association between dietary fibers intake and hs-CRP was seen among postmenopausal women in a cross-sectional study of 1958 participants (64). Our current cross-sectional study, which boasts the largest sample size to date, aligns with prior research findings.

Dietary fibers constitute essential components of human nutrition. The Institute of Medicine stipulates a daily recommended intake of 30.8 g for males aged 31–50 and 25 g for females aged 31–50 (65). However, the European Food Safety Authority advocates for a higher intake range of 25–38 g/day to mitigate risks associated with type 2 diabetes, cardiovascular disease, colorectal cancer, overweight, and obesity (66). It is evident that a significant portion of participants in this study fail to meet the recommended dietary fibers intake.

Dietary fibers have demonstrated both direct or indirect protective effects on the immune system in *in vivo*, *in vitro* and population-based study. Despite their lack of digestion or absorption in the intestinal tract, dietary fibers are regarded as vital fuel sources for gut microbiota (67). The gut microbiota, such as *Clostridium*, *Bacteroides*, *Bifidobacterium*, *Prevotella*, and *Ruminococcus* (3), can ferment dietary fibers and produce a variety of metabolites associated with immune system and inflammation, the most pivotal of which are short-chain fatty acids (SCFAs) (68). Cellular experiments have illustrated that SCFAs can function as inhibitors of histone deacetylases and as ligands for G-protein-coupled receptors and aryl hydrocarbon receptors, impacting various physiological processes including immunophysiology (69–72). Previous studies have demonstrated the ability of SCFAs to affect immune niches in the lungs, intestines, and other organs of the host. Lung dendritic cells in propionate-treated mice displayed high phagocytic capacity but impaired promotion of T helper type 2 cell effector function, owing to SCFA-induced alterations in bone marrow hematopoiesis leading to increased macrophage and dendritic cell precursors (72). Lung Type 2 innate lymphoid cells (ILC2s)-driven airway hyperreactivity and inflammation were ameliorated by systemic or intranasal SCFA butyrate administration in mice, likely through histone deacetylase inhibition suppressing ILC2 proliferation, GATA3 expression, and cytokine production; similar SCFA butyrate effects were confirmed in human ILC2s (73). For intestinal immunity, *in vitro* SCFA treatment of human intestinal epithelial cells enhances the epithelial barrier and dampens immune responses *via* increased IL-10RA (74), while SCFA binding to GPR43 on colonocytes stimulates potassium (K+) efflux and hyperpolarization, activating the NLRP3 inflammasome and protecting intestinal epithelial integrity (75). Beyond SCFAs production, recent studies suggests that dietary fibers have direct effect on the epithelial cells and immune cells in the gastrointestinal tract. *In vitro* studies show dietary fibers can directly attenuate inflammatory cytokine production from dendritic cells co-cultured with intestinal epithelial supernatants, dependent on fiber interactions with Toll-like receptors. Specific fibers differentially modulate T cell responses and regulatory T cell cytokines. β-Glucan protects intestinal epithelial barrier integrity during Salmonella infection by preserving tight junctions and limiting invasion. Additionally, some fibers elicit cytokine secretion from intestinal epithelial cells through MyD88/TLR4 signaling (76–78). These findings demonstrate dietary fiber interactions with intestinal immune and epithelial cells regulate inflammatory responses and barrier function *via* pattern recognition receptor pathways. In addition to *in vivo* and *in vitro* evidence, prospective cohort studies have indicated that the early consumption of dietary fibers may assist in decreasing the chances of allergies and asthma in adulthood (79). Likewise, high fiber maternal diets during pregnancy are linked to lower risk of allergic diseases like rhinitis and eczema in offspring (80). A cross-section study based on NHANES data conducted in adults indicates that high-fiber diet may reduce the serum CRP level and decrease odds of having asthma (10).

This study had several advantages. It pioneers the identification of the association between dietary fiber intake and systemic immune and inflammatory states, with the SII serving as the primary indicator. Leveraging a substantial and representative sample from NHANES, the study employs a comprehensive array of indicators to gauge systemic immune and inflammatory status. Nevertheless, the study had some limitations. A cross-sectional study design is incapable of determining the causality and is unable to remove the insidious residual confusing results from unmeasured or unidentified confounding factors. Despite our adjustments to DII, the confounding effects of the anti-inflammatory component of the diet such as vitamins, flavonoids, and other substances could not be completely eliminated. The study’s reliance on dietary fiber intake data from just two 24-h dietary reviews introduces a potential limitation, as dietary preferences naturally fluctuate from day to day, potentially impacting the precision of the assessment. Furthermore, short-term dietary assessments are not considered to be an accurate representation of a participant’s true dietary intake and the recall bias in dietary questionnaire was difficult to evaluate.

The current study has the following implications for future research. Our findings provide evidence for a negative correlation between dietary fibers intake and systemic immunity and inflammation biomarkers, which highlights the potential therapeutic role of dietary fibers in immune and inflammatory diseases. Therefore, well-designed randomized controlled trials or prospective cohort studies with long-term follow-up are warranted to further evaluate dietary fiber intake as an intervention or exposure, respectively. The relationship between ferritin and dietary fibers remains a matter of debate, and further exploration of their association in populations with varying disease states is essential to elucidate the nature of their relationship.

## Conclusion

Dietary fibers intake is inversely associated with systemic immune and inflammatory biomarkers in the human body. The associations persisted in the sensitivity analysis. Thus, dietary fibers should be recommended to promote immune health.

## Data availability statement

The original contributions presented in the study are included in the article/Supplementary material, further inquiries can be directed to the corresponding author.

## Ethics statement

The studies involving humans were approved by National Health and Nutrition Examination Survey. The studies were conducted in accordance with the local legislation and institutional requirements. The participants provided their written informed consent to participate in this study.

## Author contributions

XQ designed the study. XQ, CF, YL, and YJ collected the data. XQ, YL, MC, XC, and JJ analyzed the data and drafted the manuscript. JJ revised and approved the final version of the manuscript. All authors contributed to the article and approved the submitted version.

## Conflict of interest

The authors declare that the research was conducted in the absence of any commercial or financial relationships that could be construed as a potential conflict of interest.

## Publisher’s note

All claims expressed in this article are solely those of the authors and do not necessarily represent those of their affiliated organizations, or those of the publisher, the editors and the reviewers. Any product that may be evaluated in this article, or claim that may be made by its manufacturer, is not guaranteed or endorsed by the publisher.

## Footnotes

^1^ https://www.cdc.gov/nchs/nhanes/index.htm

^2^ http://www.R-project.org
