# Supplementary material for: The associations between dietary fibers intake and systemic immune and inflammatory biomarkers, a multi-cycle study of NHANES 2015–2020
Source: Front Nutr. 2023 Aug 31;10:1216445. doi: 10.3389/fnut.2023.1242115 (PMC10501836; doi:10.3389/fnut.2023.1242115)
Supplement: Supplementary file 1 [file Table_1.docx]

**Supplementary Table 1.** Sensitivity analysis for the association between dietary fibers intake and SIRI, NLR, PLR, RA, ferritin, hs-CRP, and WBC.

| **Exposure** | **Non-adjusted model, β (95%CI) P** | **Minimally-adjusted model, β (95%CI) P** | **Fully-adjusted model, β (95%CI) P** |
| --- | --- | --- | --- |
| **SIRI** |  |  |  |
| Dietary fiber | -0.00436 (-0.00759, -0.00113) 0.010699 | -0.00673 (-0.01019, -0.00328) 0.000359 | -0.00617 (-0.01049, -0.00184) 0.006536 |
| Dietary fiber tertiles |  |  |  |
| Low | Ref | Ref | Ref |
| Middle | -0.11159 (-0.15989, -0.06329) 0.000034 | -0.14427 (-0.19213, -0.09641) <0.000001 | -0.13261 (-0.18359, -0.08164) 0.000009 |
| High | -0.09372 (-0.16494, -0.0225) 0.012711 | -0.14929 (-0.22275, -0.07583) 0.000216 | -0.14271 (-0.23017, -0.05525) 0.002238 |
| P trend | -0.0457 (-0.0815, -0.0099) 0.0154 | -0.0733 (-0.1103, -0.0363) 0.0003 | -0.0729 (-0.1148, -0.0309) 0.0018 |
| **NLR** |  |  |  |
| Dietary fiber | -0.00318 (-0.00697, 0.0006) 0.104824 | -0.00515 (-0.00894, -0.00135) 0.010446 | -0.00679 (-0.0121, -0.00149) 0.017315 |
| Dietary fiber tertiles |  |  |  |
| Low | Ref | Ref | Ref |
| Middle | -0.12493 (-0.2067, -0.04317) 0.003411 | -0.1585 (-0.23399, -0.08301) 0.000141 | -0.16959 (-0.25479, -0.08439) 0.00048 |
| High | -0.08972 (-0.18044, 0.00101) 0.052408 | -0.14404 (-0.23263, -0.05545) 0.002457 | -0.18411 (-0.30101, -0.06721) 0.004239 |
| P trend | -0.0434 (-0.0880, 0.0011) 0.0610 | -0.0704 (-0.1150, -0.0257) 0.0032 | -0.0940 (-0.1530, -0.0349) 0.0038 |
| **PLR** |  |  |  |
| Dietary fiber | 0.06314 (-0.12364, 0.24992) 0.510409 | 0.0951 (-0.07954, 0.26975) 0.290747 | -0.03947 (-0.26526, 0.18631) 0.734099 |
| Dietary fiber tertiles |  |  |  |
| Low | Ref | Ref | Ref |
| Middle | -1.20485 (-5.15531, 2.74561) 0.552532 | -1.26527 (-5.11389, 2.58335) 0.522226 | -2.51697 (-6.06811, 1.03417) 0.174669 |
| High | 0.11283 (-4.32449, 4.55015) 0.96044 | 0.22403 (-4.09345, 4.54151) 0.919391 | -2.25937 (-7.02984, 2.51109) 0.360429 |
| P trend | 0.0787 (-2.1511, 2.3085) 0.9451 | 0.1383 (-2.0319, 2.3086) 0.9011 | -1.1641 (-3.5324, 1.2043) 0.3426 |
| **RA** |  |  |  |
| Dietary fiber | -0.00667 (-0.00834, -0.005) <0.000001 | -0.00586 (-0.0077, -0.00402) <0.000001 | -0.00219 (-0.00401, -0.00038) 0.019228 |
| Dietary fiber tertiles |  |  |  |
| Low | Ref | Ref | Ref |
| Middle | -0.03578 (-0.07337, 0.00181) 0.067643 | -0.03609 (-0.07224, 0.00006) 0.050244 | 0.0017 (-0.03242, 0.03582) 0.922893 |
| High | -0.12089 (-0.16076, -0.08101) <0.000001 | -0.11599 (-0.16213, -0.06984) 0.000006 | -0.046 (-0.08835, -0.00365) 0.041294 |
| P trend | -0.0609 (-0.0812, -0.0406) <0.0001 | -0.0584 (-0.0813, -0.0355) <0.0001 | -0.0224 (-0.0435, -0.0013) 0.0459 |
| **Ferritin** |  |  |  |
| Dietary fiber | 0.13235 (-0.39264, 0.65734) 0.623235 | -1.14666 (-1.54392, -0.7494) <0.000001 | -0.95361 (-1.52542, -0.3818) 0.001817 |
| Dietary fiber tertiles |  |  |  |
| Low | Ref | Ref | Ref |
| Middle | 11.31486 (-0.55118, 23.18091) 0.067159 | -3.72561 (-12.68775, 5.23652) 0.418985 | -1.31563 (-9.14028, 6.50903) 0.743955 |
| High | 12.47354 (-1.70518, 26.65226) 0.090485 | -14.19397 (-24.88198, -3.50596) 0.012073 | -7.48722 (-21.32592, 6.35147) 0.297142 |
| P trend | 6.1471 (-1.0214, 13.3156) 0.0986 | -7.1613 (-12.5938, -1.7288) 0.0126 | -3.6835 (-10.4704, 3.1035) 0.2954 |
| **hs-CRP** |  |  |  |
| Dietary fiber | -0.07589 (-0.09321, -0.05858) <0.000001 | -0.06799 (-0.08719, -0.04878) <0.000001 | -0.03717 (-0.06421, -0.01013) 0.011131 |
| Dietary fiber tertiles |  |  |  |
| Low | Ref | Ref | Ref |
| Middle | 0.05516 (-0.46647, 0.57678) 0.836609 | 0.11878 (-0.41337, 0.65093) 0.663602 | 0.25695 (-0.25625, 0.77015) 0.314445 |
| High | -1.25147 (-1.68932, -0.81361) <0.000001 | -1.11849 (-1.60724, -0.62973) 0.000042 | -0.69244 (-1.31527, -0.06961) 0.030275 |
| P trend | -0.6378 (-0.8578, -0.4177) <0.0001 | -0.5722 (-0.8178, -0.3265) <0.0001 | -0.3313 (-0.6335, -0.0291) 0.0393 |
| **WBC** |  |  |  |
| Dietary fiber | -0.03216 (-0.04035, -0.02397) <0.000001 | -0.0295 (-0.03788, -0.02113) <0.000001 | -0.01922 (-0.03244, -0.00601) 0.00757 |
| Dietary fiber tertiles |  |  |  |
| Low | Ref | Ref | Ref |
| Middle | -0.33041 (-0.6611, 0.00029) 0.055467 | -0.30269 (-0.63352, 0.02813) 0.078852 | -0.20169 (-0.48676, 0.08338) 0.175419 |
| High | -0.62203 (-0.92221, -0.32184) 0.000162 | -0.5699 (-0.87049, -0.26931) 0.000503 | -0.4249 (-0.7238, -0.126) 0.009019 |
| P trend | -0.3107 (-0.4585, -0.1629) 0.0001 | -0.2846 (-0.4325, -0.1367) 0.0004 | -0.2122 (-0.3618, -0.0625) 0.0090 |
| **Neutrophils** |  |  |  |
| Dietary fiber | -0.02017 (-0.02555, -0.01479) <0.000001 | -0.0184 (-0.0238, -0.01299) <0.000001 | -0.01282 (-0.01911, -0.00653) 0.000225 |
| Dietary fiber tertiles |  |  |  |
| Low | Ref | Ref | Ref |
| Middle | -0.21122 (-0.35126, -0.07117) 0.004643 | -0.18957 (-0.32557, -0.05357) 0.008624 | -0.13258 (-0.25923, -0.00594) 0.04871 |
| High | -0.35974 (-0.50321, -0.21626) 0.000009 | -0.3208 (-0.4598, -0.1818) 0.000037 | -0.23829 (-0.38141, -0.09517) 0.002683 |
| P trend | -0.1793 (-0.2505, -0.1081) <0.0001 | -0.1598 (-0.2289, -0.0908) <0.0001 | -0.1195 (-0.1916, -0.0473) 0.0027 |
| **Lymphocyte** |  |  |  |
| Dietary fiber | -0.01012 (-0.01552, -0.00472) 0.000548 | -0.00845 (-0.0138, -0.00309) 0.003183 | -0.00525 (-0.01475, 0.00424) 0.286148 |
| Dietary fiber tertiles |  |  |  |
| Low | Ref | Ref | Ref |
| Middle | -0.11405 (-0.38671, 0.1586) 0.41596 | -0.0982 (-0.36951, 0.17311) 0.481288 | -0.06516 (-0.30896, 0.17864) 0.604128 |
| High | -0.23812 (-0.4899, 0.01366) 0.069355 | -0.20794 (-0.46221, 0.04634) 0.115155 | -0.17188 (-0.41417, 0.07042) 0.174315 |
| P trend | -0.1191 (-0.2429, 0.0047) 0.0646 | -0.1041 (-0.2291, 0.0209) 0.1088 | -0.0854 (-0.2074, 0.0366) 0.1795 |
| **Monocyte** |  |  |  |
| Dietary fiber | -0.00121 (-0.00203, -0.00039) 0.005316 | -0.00181 (-0.00265, -0.00096) 0.000108 | -0.00121 (-0.00246, 0.00004) 0.056493 |
| Dietary fiber tertiles |  |  |  |
| Low | Ref | Ref | Ref |
| Middle | -0.00871 (-0.02461, 0.00719) 0.287912 | -0.01549 (-0.03121, 0.00023) 0.059031 | -0.00961 (-0.02578, 0.00657) 0.234188 |
| High | -0.01561 (-0.03482, 0.00359) 0.117065 | -0.02769 (-0.04748, -0.0079) 0.008389 | -0.0169 (-0.04069, 0.0069) 0.156988 |
| P trend | -0.0078 (-0.0174, 0.0018) 0.1190 | -0.0138 (-0.0238, -0.0039) 0.0089 | -0.0085 (-0.0197, 0.0028) 0.1497 |
| **Eosinophils** |  |  |  |
| Dietary fiber | -0.00054 (-0.00109, 0.00001) 0.061127 | -0.00072 (-0.0013, -0.00015) 0.016743 | 0.00002 (-0.00064, 0.00068) 0.955004 |
| Dietary fiber tertiles |  |  |  |
| Low | Ref | Ref | Ref |
| Middle | 0.00178 (-0.00977, 0.01333) 0.763627 | -0.00082 (-0.01199, 0.01036) 0.886872 | 0.00317 (-0.00824, 0.01458) 0.589993 |
| High | -0.00847 (-0.02355, 0.00662) 0.276362 | -0.01285 (-0.02849, 0.0028) 0.113708 | -0.00033 (-0.0178, 0.01715) 0.970875 |
| P trend | -0.0043 (-0.0120, 0.0033) 0.2713 | -0.0065 (-0.0145, 0.0014) 0.1131 | -0.0001 (-0.0087, 0.0085) 0.9853 |
| **Basophils** |  |  |  |
| Dietary fiber | -0.0003 (-0.00049, -0.00011) 0.003135 | -0.00027 (-0.00047, -0.00007) 0.009358 | -0.00018 (-0.00044, 0.00007) 0.151332 |
| Dietary fiber tertiles |  |  |  |
| Low | Ref | Ref | Ref |
| Middle | -0.00043 (-0.00443, 0.00357) 0.833817 | -0.00051 (-0.0046, 0.00358) 0.808844 | 0.00125 (-0.00328, 0.00578) 0.577706 |
| High | -0.00436 (-0.00957, 0.00085) 0.106892 | -0.00425 (-0.00944, 0.00095) 0.115321 | -0.00294 (-0.00869, 0.00281) 0.304413 |
| P trend | -0.0022 (-0.0048, 0.0004) | -0.0022 (-0.0048, 0.0005) 0.1114 | -0.0014 (-0.0042, 0.0014) 0.3287 |

**Abbreviations:** SIRI, systemic inflammation response index; NLR, neutrophil-to-lymphocyte ratio; PLR, platelet-lymphocyte ratio; RA, red blood cell distribution width-to-albumin ratio; hs-CRP, high-sensitivity C-reactive protein; CI, confidence interval.

**Non-adjusted model:** no covariates were adjusted. **Minimally-adjusted model:** age and gender were adjusted. **Fully-adjusted model:** age, gender, race, family monthly poverty level index, alcohol consumption, smoking status, vigorous recreational activities, body mass index level, hyperlipidemia, hypertension, diabetes, and dietary inflammatory index.
